# Distinct inflammatory biomarkers associated with rheumatological outcomes in chronic chikungunya disease

**DOI:** 10.1038/s41598-026-35570-x

**Published:** 2026-04-17

**Authors:** Lucas Sousa Magalhães, Juliana Cardoso Alves, Regina Adalva de Lucena Couto Ócea, Alejandra Debbo, Priscila Lima dos Santos, Suresh Mahalingam, Mauro Martins Teixeira, Amelia Maria Ribeiro de Jesus, Angela Maria da Silva, Roque Pacheco de Almeida, Camilla Natália Oliveira Santos

**Affiliations:** 1 Laboratório de Imunologia e Biologia Molecular, Universidade Federal de Sergipe, Aracaju, Brazil; 2 Setor de Parasitologia e Patologia, Instituto de Ciência Biológicas e da Saúde, Universidade Federal de Alagoas, Maceió, Alagoas Brazil; 3 Programa de Pós-Graduação em Ciências da Saúde, Universidade Federal de Sergipe, Aracaju, Brazil; 4 Departamento de Medicina, Hospital Universitário-EBSERH, Universidade Federal de Sergipe, Aracaju, Sergipe Brazil; 5 Departamento de Educação em Saúde, Universidade Federal de Sergipe, Lagarto, Brazil; 6 Institute for Biomedicine and Glycomics, Griffith University, Gold Coast, Australia; 7 Global Virus Network (GVN) Centre of Excellence in Arboviruses, Griffith University, Gold Coast, QLD Australia; 8 School of Pharmacy and Medical Sciences, Griffith University, Gold Coast, Australia; 9 Departamento de Bioquímica e Imunologia, Universidade Federal de Minas Gerais, Belo Horizonte, Brazil; 10 Instituto Federal de Educação, Ciência e Tecnologia Baiano – Campus Itaberaba, Itaberaba, Brazil

**Keywords:** Rheumatological manifestations, Immunological mediators, Biomarkers, Chronic infection, Chronic chikungunya arthritis., Cytokines, Infectious diseases, Virology, Biomarkers, Rheumatology

## Abstract

Chikungunya virus (CHIKV) poses an enduring public health threat in endemic regions and can progress from acute febrile illness to chronic, disabling rheumatologic disease. We performed an unmatched case–control study of 20 patients sampled during the acute phase (< 14 days) and 20 recruited ≥ 90 days after onset who met criteria for chronic chikungunya disease (CCD). At enrolment we quantified 45 serum cytokines/chemokines by multiplex assay; clinical data were recorded for all, and chronic cases were re-evaluated at 3, 12 and 24 months. CCD participants were older, reported greater initial pain and more often received symptomatic corticosteroids. Acute CHIKV infection showed a focused antiviral signature characterized by higher CCL2, CXCL10, IFN-α and IL-1RA, whereas CCD displayed a broader, non-resolving profile enriched for IL-21, GM-CSF, IL-23, LT-α, IL-4, IL-9, IL-31 and FGF-2. Over follow-up, many CCD cases improved; however, in time-to-discharge analyses, higher IL-23, baseline rheumatoid arthritis, and higher VAS were associated with persistence, whereas higher FGF-2 and IL-4, arthrosis, and anti-CHIKV IgG were associated with earlier resolution. Acute and chronic chikungunya exhibit distinct soluble-mediator signatures. A targeted early antiviral response aligns with recovery, while a broad Th2/Th17-skewed milieu aligns with chronicity. IL-23, for risk, and FGF-2/IL-4, for resolution, emerge as candidate biomarkers for risk stratification and therapeutic trials, alongside clinical factors.

**Supplementary Information:**

The online version contains supplementary material available at 10.1038/s41598-026-35570-x.

## Introduction

Chikungunya virus (CHIKV) is a re-emerging alphavirus of the family *Togaviridae*, transmitted mainly by *Aedes* mosquitoes^1^. First identified in Africa, the virus has spread widely over the past two decades, producing major epidemics, chiefly in tropical and subtropical regions that favor vector proliferation^2^.

While many patients present only self-limited fever, myalgia and rash, the arthralgia of CHIKV infection can evolve into a persistent, disabling condition now termed chronic chikungunya disease (CCD)^3^. Additional concerns include fatal outcomes^4^ and severe neurological complications^5^. The most prominent chronic sequelae are rheumatic, notably post-chikungunya inflammatory arthritis, in which dysregulated immunity drives joint inflammation^6^. When rheumatologic manifestations, such as arthralgias and arthritis, persist for more than three months, the illness meets criteria for chronic chikungunya disease^7^.

Clinical evolution depends on both host immunity and viral genotype^8^. Aberrant innate responses, marked by altered macrophage differentiation and excessive cytokines/chemokines (e.g., IL-6, CCL2), have been linked to chronicity and death^9^. Adaptive immunity also contributes diminished T-regulatory activity^10^, heightened CD8⁺ T-cell responses^11^, and a pathogenic Th17 bias^12^ all correlate with worse outcomes. CHIKV may further evade antibody neutralization, sustaining viral persistence in joints^13^. Migration of phenotypically altered myeloid cells from bone marrow^14^ and a prolonged pro-inflammatory milieu of cytokines, chemokines, and growth factors compound chronic arthritis^15^.

Despite attempts to repurpose existing agents, therapeutic CCD remains inadequate. Key gaps include reliable biomarkers of progression and targeted, disease-modifying drugs^16^. Accordingly, this study evaluates immunological markers in patients with acute and chronic chikungunya and examines longitudinal clinical outcomes in those with chronic rheumatologic manifestations.

## Patients and methods

### Ethics

This study was approved by the Ethics Committee of the Federal University of Sergipe under protocol number 1.486.302. All participants provided written informed consent after receiving verbal explanations about the research procedures from the researchers. The research followed the Declaration of Helsinki.

## Study design

This research is an observational study utilizing an unmatched case-control sampling at baseline with longitudinal follow-up for case group. The study encompassed patients with confirmed CHIKV infection who were selected from those who attended the University Hospital of the Federal University of Sergipe, in Aracaju city, Sergipe state, Brazil. Recruitment occurred throughout 2016, from January to December, and coinciding with the arbovirus epidemic in the northeastern region of Brazil^17^.

## Groups definition and sampling

In a prior study^18^, 401 patients with suspected acute arboviral infection were screened, and all underwent RT-qPCR testing for CHIKV and Zika virus (ZIKV). Of these, 97 patients were CHIKV-positive and ZIKV-negative. Concurrently, the rheumatology service at the same University Hospital, a local reference center, evaluated 25 individuals with a history of CHIKV infection who had developed CCD. Due to budgetary constraints, we enrolled 40 patients divided equally into two groups (*n* = 20 per group): Chronic-CHIKV group; patients with a prior clinical diagnosis of CHIKF who developed persistent arthralgias, joint pain, sometimes with periarticular edema, lasting more than three months after the acute phase. At recruitment, these patients exhibited no acute CHIKF signs or symptoms other than arthralgias. The 20 selected individuals were randomly selected from all attended patients at the rheumatology service. Acute-CHIKV group; consecutive patients presenting within 14 days of symptom onset who tested positive by molecular diagnosis. These 20 individuals were simply randomly selected from all laboratory-confirmed acute cases, as explained previously, and that did not evolve to CCD (evaluated after three months of disease onset). Additionally, uninfected individuals with no clinical symptoms and negative anti-CHIKV serology were enrolled as immunological controls. They were recruited from the same geographic area as the patients, and their age and sex distributions mirrored those of the CHIKV groups.

Exclusion criteria included patients with cancer or those diagnosed with other inflammatory or infectious diseases. Due to the high incidence of ZIKV circulation during the study period^17^, those testing positive were excluded. The study included adult patients (over 18 years of age).

After patients’ consent, data from the initial disease manifestation were retrieved for both groups (at baseline point) and included age, sex, municipality of origin, type and duration of symptoms during the acute phase (fever, exanthema, conjunctivitis, myalgia, retro-orbital pain, and lymphadenopathy). Medication use and the presence of comorbidities were also recorded. Pain intensity was assessed using a visual analogue scale (VAS) ranging from 0 to 10^19^. The primary outcome was established as cure from chikungunya disease, assessed by clinicians in the absence of signals and symptoms. Biological samples were collected from all participants at the time of enrollment in the study (baseline point) and stored in the freezer (-80 °C) until assay execution for serological analysis of specific antibodies and immunological markers. None of the laboratory staff or clinicians were blinded during the patients assessing.

## Longitudinal assessment of patients with CCD

A 24-month longitudinal study was conducted in patients with CCD. Each participant underwent in-person evaluations at 3, 12, and 24 months after enrollment (≥ six months after disease onset in the first evaluation). At every visit, a specialist clinically assessed several domains, including rheumatological manifestations, in accordance with national guidelines^19^: inflammatory arthritis, tendinitis, plantar fasciitis, arthrosis, carpal tunnel syndrome, arthralgias, spinal disorders, fibromyalgia, and alopecia. Additional evaluations included potential clinical complications such as infectious, vascular, neurological, and neoplastic conditions. Disease activity and pain were quantified at each visit using the Disease Activity Score 28 (DAS28) and Visual Analogue Scale (VAS), respectively.

Treatment protocols varied and included NSAIDs, analgesics, opioids, corticosteroids, methotrexate, chloroquine, pregabalin, antiarthritic drugs, and antidepressants, all administered according to the chikungunya disease treatment guidelines set forth by the Brazilian Society of Rheumatology^20^.

For longitudinal analysis, we defined the following outcomes: the primary outcome was discharge from clinical follow-up, determined by the rheumatologist based on the absence of persistent rheumatological symptoms and no further requirement for ongoing treatment (according to national recommendations). Secondary outcomes included persistent rheumatological conditions, loss to follow-up (patients who did not return), and diagnosis of an unrelated disease during the follow-up period.

## Molecular detection of chikungunya virus

For virus detection, viral RNAs were extracted from serum or plasma samples using the QIAamp Viral RNA Mini Kit (QIAGEN, Germany), following the manufacturer’s instructions. Reverse transcription followed by quantitative real-time polymerase chain reaction (RT-qPCR) was conducted using Invitrogen’s SuperScript^®^ III Platinum^®^ One-Step RT-qPCR System (Thermo Fisher Scientific, USA). Primers and probes specifically designed for detecting CHIKV^21^ and ZIKV^22^ viruses were obtained from Integrated DNA Technologies (IDT, USA). Amplification was carried out on an ABI 7500 FAST system (Applied Biosystems, USA). Samples with a cycle threshold (Ct) value of 38 or lower were considered positive for CHIKV or ZIKV virus infection^21^.

### Serology analysis for CHIKV antibodies

Serological assays were performed using Anti-Chikungunya Virus ELISA IgM and IgG kits (Euroimmun, Germany), following the manufacturer’s recommended procedures. Results were obtained using a BioTek Epoch microplate reader (Agilent Technologies, USA) and evaluated semi-quantitatively by calculating a ratio of the extinction value, as recommended by the manufacturer.

## Serum cytokines, chemokines, and growth factors quantification

The levels of cytokines, chemokines, and growth factors were assessed using the ProcartaPlex™ Human Cytokine/Chemokine/Growth Factor Panel 1, 45-plex kit (Thermo Fisher Scientific, USA). The assay was conducted according to the manufacturer’s instructions using a Luminex^®^ 100 system and xPONENT^®^ 3.1 software (Luminex Corporation, USA). Results are expressed in picograms per milliliter (pg/mL).

The analytes included in the study are categorized by manufacturer as: **Th1/Th2**: granulocyte-macrophage colony-stimulating factor (GM-CSF), interferon γ (IFN-γ), interleukin 1 β (IL-1β), IL-2, IL-4, IL-5, IL-6, IL-8, IL-12p70, IL-13, IL-18, tumor necrosis factor α (TNF-α); **Th9/Th17/Th22/Treg**: IL-9, IL-10, IL-17 A, IL-21, IL-22, IL-23, IL-27; **Inflammatory cytokines**: IFN-α, IL-1α, IL-1RA, IL-7, IL-15, IL-31, LIF, Lymphotoxin alpha (LT-α) (formerly known as TNF-β); **Chemokines**: C-C motif chemokine ligand 11 (CCL11, also known as Eotaxin), C-X-C motif chemokine ligand 1 (CXCL1), CXCL10 (formerly known as interferon-γ-induced protein 10 kDa, IP-10), CCL2 (known as monocyte chemoattractant protein-1, MCP-1), CCL3 (also known as macrophage inflammatory protein alpha, MIP-1α), CCL4 (also known as MIP-1β), CCL5 (known as regulated on activation, normal T cell expressed and secreted, RANTES), CXCL12 (known as stromal cell-derived factor 1α, SDF-1); **Growth factors**: brain-derived neurotrophic factor (BDNF), epidermal growth factor (EGF), fibroblast growth factor 2 (FGF-2, also known as FGF-β), hepatocyte growth factor (HGF), nerve growth factor (NGF), platelet derived growth factor subunit B (PDGF-BB), placental growth factor 1 (PIGF-1), stem cell factor (SCF), vascular endothelial growth factor A (VEGF-A), VEGF-D.

## Data processing and statistical analysis

All collected data were stored in Microsoft Excel for Microsoft 365. Statistical analysis was conducted using GraphPad Prism 9 and R Studio version 4.3. A 95% confidence level was established for all analyses, which were performed with two-tailed tests. The Gaussian distribution was assessed using the Shapiro-Wilk test. Categorical variables are presented as counts and percentages of the group; Fisher’s exact test was used with effect size measured by the odds ratio. Continuous variables are presented as median plus interquartile range [25%–75%], and Mann-Whitney or Kruskal-Wallis tests were followed by an adjusted Dunn test for multiple comparisons. Spearman’s correlation (rho) test was employed for correlation among variables.

For the analysis of immunological markers, principal component analysis (PCA) was performed using the ‘factoextra’ package and plotted with the ‘ggfortify’ package. A volcano plot was created using GraphPad Prism after z-score determinations and log conversion. Receiver Operating Characteristic (ROC) curves were also generated using GraphPad Prism, with Youden’s index used to find the optimized values and markers (two-sided test, threshold > 0.50). Correlation matrices of cytokines were generated using the ‘corrplot’ package in R, displaying only significant correlations. Biological relationship complementary analysis was conducted using the STRING Core Data Resource^23^, showing medium-confidence interactions among immunological markers (score ≥ 0.400). For the STRING enrichment analysis, the measure of effect is shown as strength [Log₁₀(observed/expected)], and the adjusted p-value is obtained using the Benjamini–Hochberg procedure.

For regression and survival analyses we used R, employing the survival and survminer packages together with base functions. A linear probability model (Group ~ Age + VAS) quantified the association between group status and numerical predictors. In the longitudinal CCD anlaysis, two Cox proportional-hazards models were fitted: (i) a clinical model, Surv(time, status) ~ Tendinitis + Plantar Fasciitis + Arthralgia + Inflammatory Arthritis + Arthrosis + Carpal Tunnel Syndrome + Spinal Disorder + Fibromyalgia + DAS28 + VAS + IgM + IgG — and (ii) an immunological model, Surv(time, status) ~ IL-4 + GM-CSF + IL-9 + LT-α + IL-23 + IL-21 + FGF-2 + IL-31. “Time” (days) was measured from disease start to each rheumatology assessment, and “status” indicated discharge (cure) versus any competing outcome.

## RESULTS

### Patients with CCD display more intense initial disease

As shown in Table 1, Median [IQR] age was 51 [41–61] years in the CCD group versus 34.5 [21–49] years in the Acute group (*p* = 0.001). Both groups had a similar sex distribution (85% female in Chronic vs. 80% female in Acute; *p* > 0.999). The duration of symptoms was 135 [90–210] days in Chronic versus 3 [1–4] days in Acute (*p* < 0.0001). Although the frequency of arthralgia was very similar in both groups, the CCD patients reported more intense pain: VAS pain 9 [8–9] versus 8 [7–9] in Acute (*p* = 0.001). A linear probability model (Group ~ Age + VAS) confirmed that both Age (β = 0.013, SE = 0.0045; *p* = 0.002) and VAS (β = 0.131, SE = 0.0519; *p* = 0.010) were independently associated with membership in the Chronic group (full results in Supplementary Table 1).

**Table 1:** Clinical and epidemiological information from patients included in the study.

Variables	Chronic	Acute	*p*-value ^A^	Effect size β or OR [95% CI]
	*n* = 20 (%)	*n* = 20 (%)		
*Age * ^*B*^				
Age, years	51 [41–61]	34.5 [21–49]	**0.001**	0.013; *p* = 0.002 ^C^
Sex (%)				
Female	17 (85)	16 (80)	> 0.999	*ns*
Time of symptoms				
From start to sampling, days	135 [90–210]	3 [1–4]	**< 0.0001**	- ^D^
Laboratorial analysis				
IgM anti-chikungunya	9 (45)	8 (40)	**-**	*-*
IgG anti-chikungunya	14 (70)	NA	**-**	*-*
*Initial rheumatologic evaluation*				
Arthralgia ^E^	20 (100)	19 (95)	> 0.999	*ns*
Single	16 (80)	15 (79)	> 0.999	*ns*
Oligo	4 (20)	4 (21)	> 0.999	*ns*
Poly	0	0	-	-
Visual analog scale ^F^	9 [8–10]	8 [7–9]	**0.001**	0.131; *p* = 0.010 ^C^
Previous rheumatologic conditions	14 (70)	2 (10)	**0.0002**	21 [3.59–103.3]
*Other initial symptoms*				
Fever	18 (90)	19 (95)	> 0.999	*ns*
Exanthema	16 (80)	9 (45)	**0.048**	4.89 [1.23–16.56]
Conjunctivitis	4 (20)	8 (40)	0.300	*ns*
Myalgia	20 (100)	16 (80)	0.341	*ns*
Retro-orbital pain	5 (25)	13 (65)	**0.024**	0.18 [0.04–0.67]
Lymphadenopathy	3 (15)	3 (15)	> 0.999	*ns*
Medication in acute phase				
Antihistaminic	7 (35)	2 (10)	0.127	*ns*
NSAIDs	14 (70)	6 (30)	**0.025**	5.44 [1.48–20.6]
Analgesic	12 (60)	14 (70)	0.741	*ns*
Antipyretic	8 (40)	10 (50)	0.751	*ns*
Corticosteroid	17 (85)	3 (15)	**< 0.0001**	32.11 [5.28–137.8]
Antidiabetics	1 (5)	1 (5)	> 0.999	*ns*
Antihypertensive	4 (20)	1 (5)	0.341	*ns*
*Other chronic conditions*				
Rhinitis	5 (25)	3 (15)	0.694	*ns*
Asthma	0	0	-	-
Dermatitis	0	0	-	-
Urticaria	0	0	-	-
Systemic arterial hypertension	9 (45)	2 (10)	**0.031**	7.36 [1.28–36.99]
Diabetes mellitus	6 (30)	0	**0.020**	+*infinity*
Dyslipidemia	6 (30)	1 (5)	0.091	*ns*
Hypothyroidism	0	0	**-**	-
Depression	0	0	**-**	-

A, Data tested with: Fisher`s exact test (for association comparisons); Mann-Whitney test for continuous variables).

B, data showed in median plus interquartile range [25%-75%].

C, Details of multiple linear regression are presented in Supplementary Table 01.

D, days of symptoms was not included in regression analysis.

E, Visual analog scale of the pain ranging from 0 (no pain) to 10 (worst pain).

F, Evaluation grouped as number of affected joints: single (1 joint), oligo (2 to 4 joints), poly (5 or more).

Bold indicates statistical significative differences (*p* < 0.05). NA, indicates not evaluated for this group of patients

Clinical features in the initial phase also differed: exanthema occurred in 80% of CCD versus 45% of Acute (*p* = 0.048), whereas retro-orbital pain was more common in Acute 65% vs. 25% in Chronic (*p* = 0.024). Hypertension was present in 45% of CCD vs. 10% of Acute (*p* = 0.031), and diabetes mellitus in 30% of CCD vs. 0 of Acute (*p* = 0.020). During the acute phase, NSAID use was more frequent in CCD 70% vs. 30% in Acute (*p* = 0.025), and corticosteroid use likewise 85% vs. 15% (*p* < 0.0001).

Serology results (CCD group only): among 20 Chronic patients, 70% were IgG-positive and 45% were IgM-positive (all IgM + were also IgG+). In the Acute group, 40% were IgM + at baseline (no acute IgG testing). Within Chronic patients, IgM + versus IgM– subsets did not differ in median VAS or symptom duration.

### CCD is related to robust but non-resolving immune response profile

**Fig. 1:**
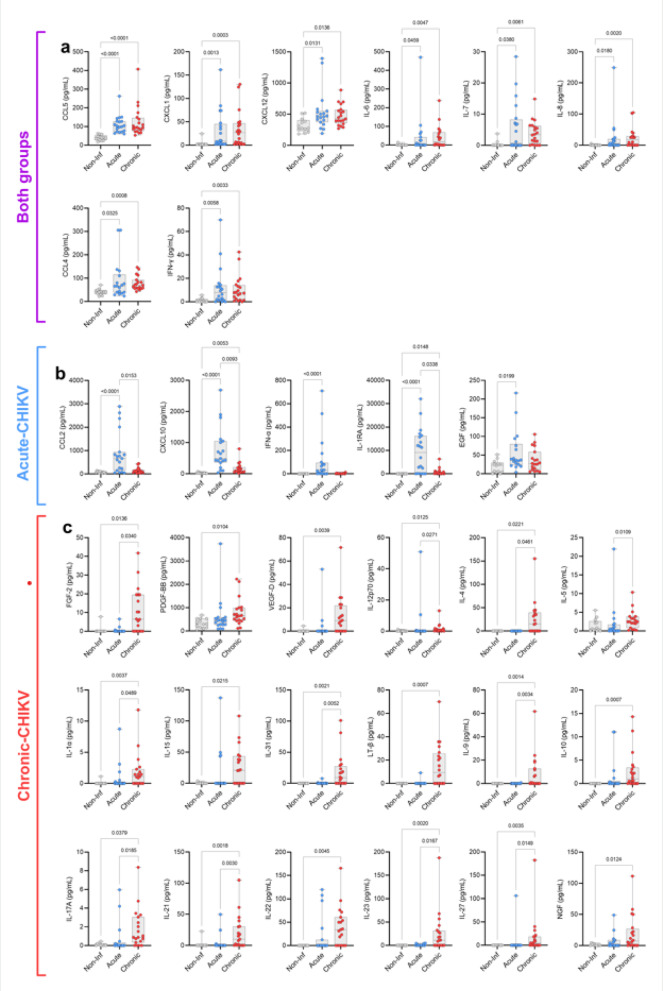
Cytokines, chemokines, and growth factors quantified in the sera of people with Acute- (blue) and Chronic- (red) chikungunya disease and non-infected donors (gray). (**a**) Results showing differential mediators in both groups, Acute and Chronic, compared to non-infected controls; Mediators with higher levels in Acute groups when compared to Chronic group and/or non-infected donors (**b**); Results showing differential mediators in Chronic group compared with Acute and Non-infected groups (**c**). Each dot represents a person, boxplots show median plus 25% and 75% interquartile range and error bars show minimum and maximum values. Comparisons made by Kruskal-Wallis followed by Dunn test (adjusted p-values).

We measured 45 cytokines, chemokines, and growth factors in serum from 20 Acute-CHIKV patients, 20 Chronic-CHIKV patients, and 13 age- and sex-matched non-infected controls (median age 39 years [range 31–46], 70% women). Compared to controls, 32/45 mediators were differentially expressed in at least one patient group. Of these, eight mediators (CCL5, CXCL1, CXCL12, IL-1RA, IL-6, IL-7, IL-8, IFN-γ) were elevated in both Acute and Chronic groups. (Fig. 1a).

Within the CHIKV cohorts, five markers (CCL2, CXCL10, IFN-α, IL-1RA, and EGF) were significantly higher in Acute-CHIKV than in Chronic-CHIKV or controls (Fig. 1b). Eighteen mediators, IL-4, IL-5, GM-CSF, CCL4, VEGF-D, FGF-2, PDGF-BB, IL-1α, IL-15, IL-31, LT-α, IL-10, IL-27, IL-9, IL-21, IL-22, IL-23, and IL-17 A, were uniquely elevated in Chronic-CHIKV compared to both Acute and controls (Fig. 1c). The remaining markers did not differ among groups (Supplementary Fig. 2).

**Fig. 2:**
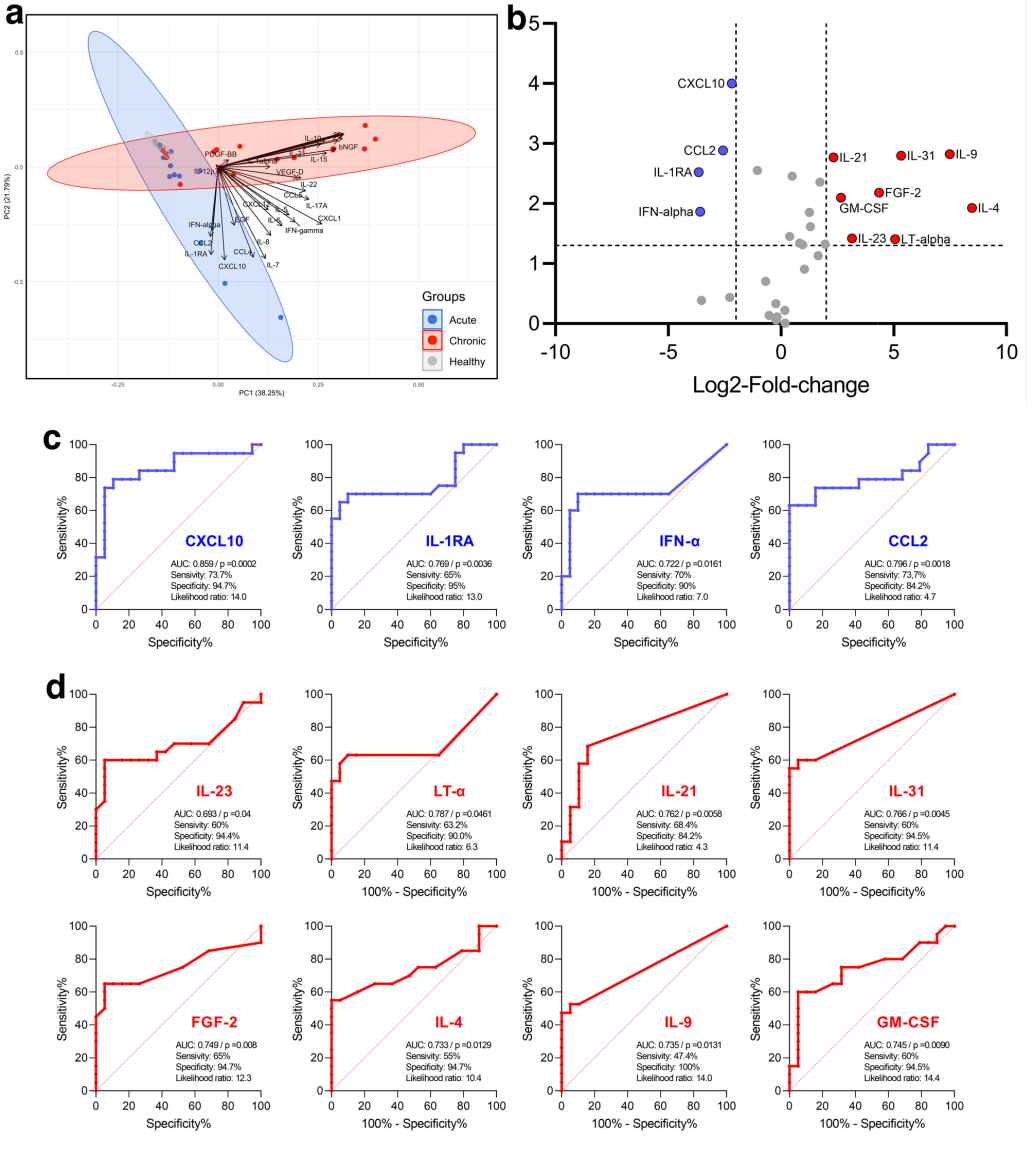
(**a**) Biplot of the principal component analysis of the 33 immune mediators with higher levels, illustrating two major distinct groups – Acute- and Chronic-chikungunya – each associated with different cytokines, chemokines, and growth factors distributed in opposite directions (loadings). (**b**) Volcano plot comparing the levels of immune mediators between people with Acute and Chronic chikungunya infection. The vertical dotted line represents the 2-fold change threshold for differences between groups, and the horizontal dotted line marks the p-value threshold. Differences were assessed using the Mann-Whitney test. (**c**) ROC curves of the CXCL10, IL-1RA, IFN-α, and CCL2. (**d**) ROC curves for all differential mediators for Chronic group, including IL-23, LT-α, IL-21, IL-31, FGF-2, IL-4, IL-9, GM-CSF. All ROC curves include the area under the curve (AUC), *p-value*, sensitivity, specificity, and the likelihood ratio for the optimal concentration cutoff. The order of each graph in both groups was established according to Youden’s index.

To visualize overall patterns, we performed principal component analysis (PCA). Acute-CHIKV patients clustered with moderate elevations in CXCL10, CCL2, CCL4, IL-1RA, EGF, and IFN-α, whereas Chronic-CHIKV patients clustered with higher levels of β-NGF, IL-15, IL-9, IL-21, IL-23, IL-1α, and VEGF-D (Fig. 2a). A volcano plot (Fig. 2b) confirmed that IFN-α, IL-1RA, CCL2, and CXCL10 were significantly upregulated in Acute versus Chronic, while GM-CSF, IL-4, IL-9, IL-21, IL-23, IL-31, LT-α, and FGF-2 were upregulated in Chronic versus Acute.

All differentially expressed mediators were evaluated as classifiers. CXCL10 best distinguished patients with Acute disease from that with CCD (AUC = 0.859; *p* = 0.0002; Youden’s J = 0.68) (Fig. 2c). Other Acute-associated markers also achieved AUCs > 0.75 (all *p* < 0.010). Among CCD-associated mediators, IL-23 (AUC = 0.693; *p* = 0.040; Youden’s J = 0.62) and FGF-2 (AUC = 0.749; *p* = 0.008; Youden’s J = 0.59) provided discrimination between groups (Fig. 2d), together with other immunological makers. No marker differentiated IgM-positive from IgM-negative CCD patients (all *p* > 0.050).

**Fig. 3:**
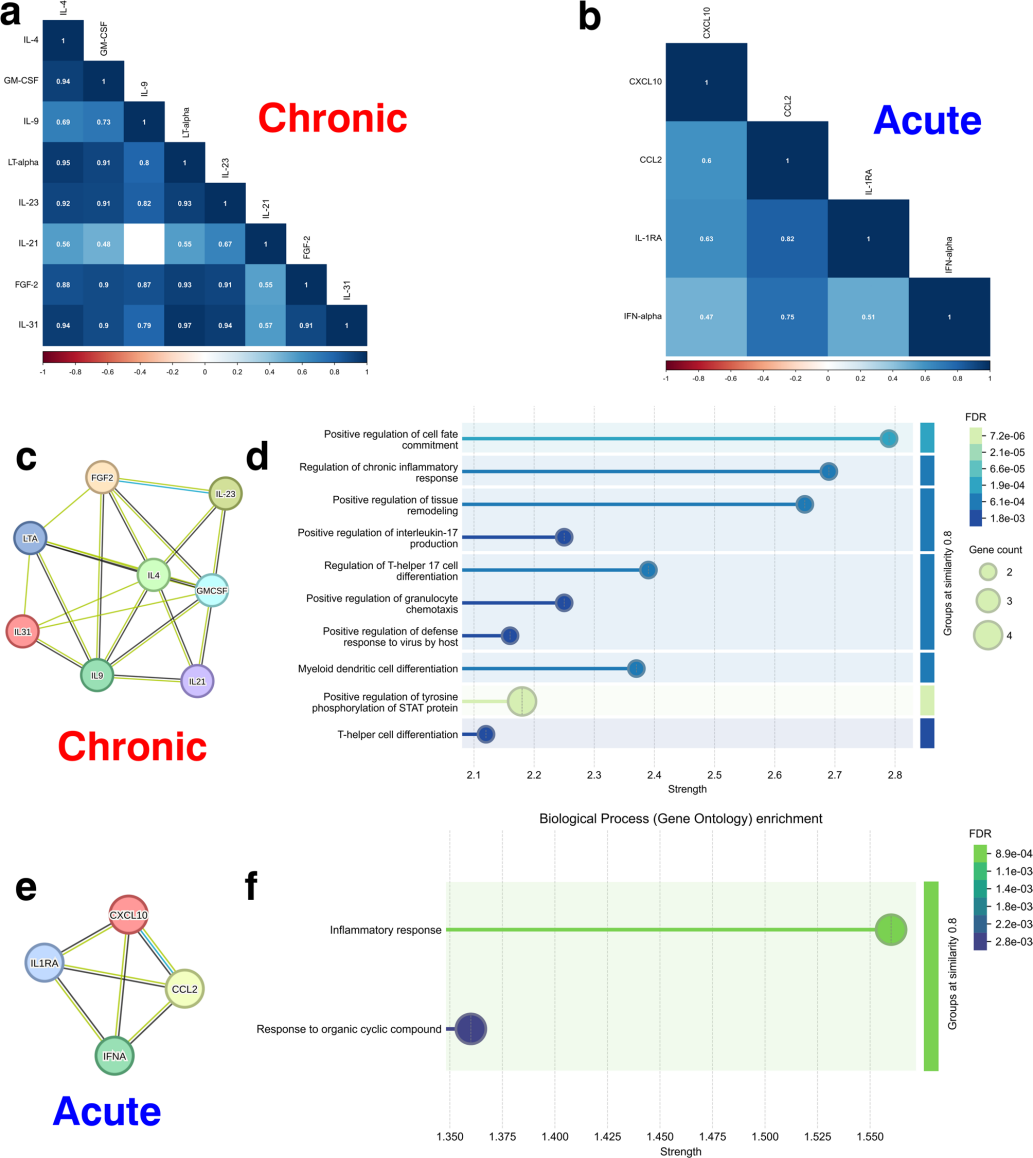
Panels (**a**) and (**b**) present correlation analyses of the four mediators with the highest circulating levels that significantly differentiate between the Acute and Chronic groups, respectively. Correlations were tested using the Spearman rank test. (**c**-**f**) Complementary analyses of protein-protein interaction and biological enrichment. (**c**) (**e**) Protein-protein interaction in Chronic- and Acute-CHIKV patients. Black lines indicate co-expressed proteins, yellow lines represent text-mining evidence, and blue lines denote evidence from curated databases. (**d**) (**f**) Enrichment analysis of biological processes were selected by the strength of enrichment together the false discovery rate (*p < 0.05*) and signal (evidence of association among proteins).

We next examined interrelationships among the top mediators. In Chronic-CHIKV, seven of eight key mediators (IL-4, GM-CSF, IL-9, IL-21, IL-23, IL-31, and LT-α) showed strong positive correlations (ρ ≥ 0.70; *p* < 0.001), whereas IL-21 correlated moderately (ρ ≈ 0.55; *p* = 0.012) (Fig. 3a). In Acute-CHIKV, the four signature mediators (IFN-α, IL-1RA, CCL2, and CXCL10) exhibited uniformly moderate correlations (ρ ≈ 0.45–0.60; *p* < 0.01) (Fig. 3b).

STRING protein–protein interaction analysis confirmed that all eight CCD-associated mediators form a biologically interconnected network, co-expression (black lines), text-mining (yellow lines), and curated database links (blue lines) densely link IL-4, GM-CSF, IL-9, IL-21, FGF-2, and IL-23 (Fig. 3c). Enrichment analysis revealed overrepresentation of “positive regulation of cell fate commitment” (strength = 2.79; *p* = 0.003), “regulation of chronic inflammatory response” (strength = 2.69; *p* = 0.004), “positive regulation of tissue remodeling” (strength = 2.65; *p* = 0.004), and “regulation of T-helper 17 cell differentiation” (strength = 2.39; *p* = 0.008) in Chronic (Fig. 3d). In contrast, the Acute network supported “inflammatory response” (strength = 1.56; *p* = 0.009) and “response to organic cyclic compounds” (strength = 1.34; *p* = 0.0289) (Fig. 3f), with a strong correlation among the four differentially expressed cytokines (Fig. 3e).

### Biomarkers of chronic disease are associated with CCD

All 20 Acute-CHIKV patients were discharged after their initial evaluation and did not develop chronic symptoms (confirmed by telemedicine after three months of disease onset). A total of 20 CCD patients were enrolled in a longitudinal analysis. In the Chronic-CHIKV group, one patient was lost to follow-up, leaving 19 individuals for re-evaluation at three months post-recruitment (≥ 6 months of disease). Detailed clinical data and treatments of these patients with CCD are presented in Table 2 and summarized in the following text. Per national guidelines, five of these 19 were discharged at three months re-evaluation due to clinical improvement; the remaining 14 continued to receive standard treatment. These persistent-symptom patients exhibited ongoing arthralgias, tendinitis, spinal disorders, fibromyalgia, or rheumatoid arthritis, similar to their initial presentations. Only one (5.3%) tested positive for rheumatoid factor (RF), and three (15.9%) had elevated C-reactive protein, including the RF-positive patient.

**Table 2:** Longitudinal clinical evaluation of patients with chronic chikungunya disease during the rheumatological follow-up

Variables	Baseline evaluation ^A^		12-months evaluation		24-months evaluation	
	*n* = 19 (%)		*n* = 14 (%)		*n* = 6 (%)	
Outcome ^B^						
Discharged	5 (26.3)		8 (57.1)		3 (50)	
Diseased	14 (74.7)		6 (42.9)		3 (50)	
Rheumatological conditions						
Rheumatoid arthritis	5 (26.3)		5 (35.7)		5 (83.3)	
Tendinitis	9 (47.4)		6 (42.9)		2 (33.3)	
Plantar fasciitis	3 (15.8)		2 (14.3)		1 (16.7)	
Arthrosis	1 (5.3)		1 (7.1)		0	
Carpal tunnel syndrome	1 (5.3)		1 (7.1)		1 (16.7)	
Arthralgias	18 (94.7)		13 (92.9)		5 (83.3)	
Spinal disorders	6 (31.6)		5 (35.7)		2 (33.3)	
Fibromyalgia	5 (26.3)		4 (28.6)		1 (16.7)	
Alopecia	0		0		0	
Clinical complications						
Infectious	1 (5.3)		1 (7.1)		1 (16.7)	
Vascular	1 (5.3)		1 (7.1)		1 (16.7)	
Neurological	0		0		0	
Neoplastic	0		0		0	
Treatment during chronic phase ^C^						
NSAIDs	6 (31.6)		4 (28.6)		1 (16.7)	
Analgesics	19 (100)		14 (100)		6 (100)	
Opioids	7 (36.8)		6 (42.9)		3 (50)	
Corticosteroids	18 (94.7)		14 (100)		6 (100)	
Methotrexate	1 (5.3)		1 (7.1)		1 (16.7)	
Chloroquine	6 (31.6)		5 (35.7)		4 (66.7)	
Pregabalin	4 (21.0)		4 (28.6)		1 (16.7)	
Antiarthritic	1 (5.3)		1 (7.1)		0	
Antidepressive	4 (21.0)		4 (28.6)		1 (16.7)	
Rheumatological scores						
Disease activity score 28	3.1 [2.9–3.4]		3.0 [2.8–3.5]		1.3 [0.8–2.8] ^#^	
Visual analogue scale	9 [8–10] ^#^		6 [4–7]		4 [3–5]	

A, Baseline evaluation made 3-months after first sampling and grouping analysis.

B, Outcome for each timepoint that lead patients to discharge or continues in follow-up.

C, treatments were made by clinician in rheumatology follow-up according to national recommendations.

#Tested with Kruskal-Wallis followed by Dunn`s test (adjusted p-value). DAS-28, *p <* 0.01 (48-months compared Baseline and 12-months); VAS, *p* < 0.001 (Baseline compared to 12- and 48-months).

At the 12-month re-evaluation (*n* = 14; ≥15 months of disease), eight additional patients were discharged after improvement, while six continued to experience chronic rheumatologic conditions; notably, the proportion of rheumatoid arthritis increased. By the 24-month follow-up (≥ 27 months of disease), those six were re-assessed: three remained symptomatic with fibromyalgia and rheumatoid arthritis, and three were discharged as clinically cured. The three patients with ongoing rheumatologic disease continue under rheumatology care with recommended medications (one with fibromyalgia and two with rheumatoid arthritis).

To assess factors associated with persistent chronicity, we performed two Cox proportional-hazards analyses: one using clinical characteristics and another using immunological biomarkers (Table 3). The clinical model was significant (likelihood ratio = 22.61 on 11 df; *p* = 0.02), as was the biomarker model (likelihood ratio = 28.10 on 8 df; *p* = 0.0004). In both models, elevated IL-23, presence of rheumatoid arthritis, and higher baseline VAS scores were associated with a significantly increased hazard of ongoing chronic arthritis. Conversely, higher FGF-2 and IL-4 levels, a diagnosis of arthrosis, and positive IgG anti-CHIKV serology were each associated with a protective effect, associated with earlier discharge from chronic disease.

**Table 3:** Evaluation of association among clinical parameters and immunological biomarkers with persistent chronic chikungunya disease overtime

Predictors	Coefficient	Hazard Ratio [95%CI]	Std. Error	Z-value	p-value	
*Clinical evaluation * ^*A*^						
Tendinitis	-0.50	0.60 [0.08–4.27]	0.99	-0.51	0.613	
Plantar fasciitis	2.54	12.68 [0.76–209.5]	1.43	1.78	0.759	
Arthralgias	29.22	4.9 × 10^12^ [*0-infinite*]	1.98 × 10^4^	0.01	0.999	
Rheumatoid arthritis	**-5.05**	**0.006 [0.0001-0.23]**	**1.84**	**-2.74**	**0.006**	
Arthrosis	**5.03**	**152.8 [1.69-1.37 × 10** ^**4**^ **]**	**2.30**	**2.19**	**0.028**	
Tunnel carpal syndrome	14.36	0.58 × 10^6^ [*0-infinite*]	1.98 × 10^4^	-0.001	0.999	
Spinal disorders	2.56	12.95 [0.54–308.6]	1.62	1.58	0.113	
Fibromyalgia	0.32	1.38 [0.14–13.3]	1.16	0.279	0.780	
DAS28	-0.39	0.68 [0.17–2.69]	0.70	-0.55	0.581	
Visual analogue scale	**-1.83**	**0.16 [0.03–0.71]**	**0.76**	**-2.40**	**0.016**	
IgM anti-CHIKV	-1.36	0.26 [0.02–2.79]	1.22	-1.11	0.264	
IgG anti-CHIKV	**5.62**	**277.0 [2.37-3.2 × 10** ^**4**^ **]**	**2.43**	**2.32**	**0.020**	
Immunological mediators ^A^						
IL-4	**0.18**	**1.19 [1.04–1.37]**	**0.07**	**2.53**	**0.011**	
GM-CSF	-0.32	0.82 [0.50–1.04]	0.19	-1.74	0.082	
IL-9	-0.09	0.91 [0.76–1.08]	0.09	-1.09	0.277	
LT-α	0.04	1.05 [0.82–1.32]	0.12	0.38	0.706	
IL-23	**-0.15**	**0.86 [0.74–0.99]**	**0.07**	**-2.12**	**0.033**	
IL-21	-0.02	0.98 [0.87–1.11]	0.06	-0.29	0.769	
FGF-2	**0.48**	**1.61 [1.11–2.33]**	**0.19**	**2.52**	**0.011**	
IL-31	0.04	0.96 [0.88–1.04]	0.04	-1.09	0.277	

A, two Cox survival regression analysis were performed for each group of variables (clinical and immunological) evaluating time of disease until patient discharge (primary outcome). Likelihood ratio test for each Cox regression was: 23.73 on 12 degrees of freedom, *p* = 0.022 (for clinical parameters) and 28.1 on 8 degrees of freedom, *p* = 0.0004 (for immunological parameters). Some values are *infinite* or have higher scale (sample limitation).

95%CI, 95% confidence interval; Std. Error, standard error.

## Discussion

Chikungunya virus (CHIKV) infection spans a clinical spectrum that ranges from asymptomatic or mild illness to severe disease characterized by debilitating rheumatologic manifestations, intense pain, and neurological complications^2^. This study advances current knowledge by detailing the clinical and immunological profiles of chikungunya patients, with particular emphasis on the chronic phase. We show that individuals who evolve to chronic chikungunya disease (CCD) are typically older, have more comorbidities, and experience a more severe acute episode than those who recover fully. Moreover, patients with CCD mount a broader, largely non-specific inflammatory response, displaying biomarkers linked to both unfavorable and favorable outcomes.

Consistent with earlier studies^24–26^, we found that age, sex, and initial disease severity influence progression to chronicity. Age is a well-recognized modifier of immunity through immunosenescence^27^. Hence, while we cannot completely disentangle age from disease stage, our findings reinforce the concept that age-related immune remodeling acts in concert with CHIKV-specific processes to drive persistent joint inflammation. Both groups were predominantly composed of women, which may reflect selection bias due to spontaneous enrollment, as explained previously^18^. Similarly, earlier studies have reported a predominance of women in rheumatoid arthritis cohorts^28^. Nevertheless, epidemiological data indicate that most arbovirus cases in Brazil occur in women^29^.

Patients with CCD reported higher pain scores during the acute phase and used anti-inflammatory medications, including corticosteroids, more frequently than their counterparts. While this likely reflects more severe initial symptoms, early corticosteroid use might also impair viral clearance^30^. Indeed, national guidelines recommend anti-inflammatories principally for the chronic stage^20^. Patients with chronic disease are also more often presented with hypertension, diabetes, or pre-existing rheumatologic conditions, comorbidities known to worsen outcomes and increase hospitalization risk^31^.

Analysis of circulating mediators further distinguished acute and chronic phases. Acute-phase patients exhibited elevated CCL2, CXCL10, IL-1RA, and IFN-α, forming an antiviral signature enriched for responses to organic cyclic compounds. CXCL10^33^ and IFN-α promote viral control^33^, whereas IL-1RA mitigates IL-1–driven inflammation and correlates with milder dengue presentations^34^. Murine data likewise implicate the CCL2/CCR2 axis in protecting against CHIKV-induced arthritis^35^. Collectively, these findings suggest that an early, well-coordinated antiviral response favors complete recovery.

By contrast, Chronic-CHIKV patients displayed a “cytokine storm”–like profile, with heightened Th1, Th2, Treg, and Th17 mediators, including GM-CSF, IL-9, and IL-4, cytokines previously associated with persistent arthralgia^36–38^. We newly identify increased lymphotoxin-α and IL-31, cytokines implicated in rheumatoid arthritis^39^ and autoimmune skin disease^40^, supporting a dysregulated inflammatory milieu. Elevated IL-21, IL-23, and (to a lesser extent) IL-17 A reinforce the importance of the Th17 axis in chronic pathology. The Th17 axis has been demonstrated as an important factor in several inflammatory and chronic diseases, including CHIKV infection^11,12^.

Regression and ROC analyses highlighted opposing roles for FGF-2 and IL-23. FGF-2 is related to tissue remodeling and repair. In models of tissue infection, FGF-2 has been shown to lead to infection control and prevent severe damage^41,42^. Conversely, IL-23 promotes an inflammatory microenvironment, which has been previously associated with infectious reactive arthritis models^43^. Their simultaneous elevation suggests a tug-of-war between reparative and destructive processes in chronic joints, a hypothesis warranting experimental confirmation.

Longitudinal follow-up underscored the diverse rheumatologic sequelae of CCD^44^. Despite persistent symptoms in some individuals, many achieved clinical remission with appropriate management, as reflected by declining pain and disease-activity scores prior to full resolution. Rheumatoid arthritis (RA) diagnosis, however, conferred a higher risk of chronic persistence; two RA patients remain symptomatic at the time of writing. Given the autoimmune nature of RA^46^, CHIKV infection may act as a trigger in susceptible hosts, underscoring the urgent need for repurposed or novel therapies for CCD^16^. Interestingly, persistent CHIKV-specific IgM in CCD patients was not associated with arthritis activity, as IgM + and IgM– subsets showed no differences in VAS scores or symptom duration. This suggests that residual IgM likely reflects long-lasting or nonspecific serological responses rather than active joint inflammation, similar to IgM-RF in RA^47^.

This single-center study had a modest sample size, increasing the risk of type II error; some true differences may be undetected. We did not have matched acute-phase sera from the same CCD patients, which limits within-person causal inference. Age differed between groups and, despite multivariable adjustment, residual confounding by age, medication use (notably early corticosteroids), comorbidities, or unmeasured factors may persist. Serum mediators may not fully mirror intra-articular biology. Finally, findings may not generalize beyond our population, which may have distinct genetic or exposure backgrounds^47,48^.

In conclusion, CCD is associated with older age, comorbidities, more intense early pain, and early anti-inflammatory use. Patients who recover show a strong, targeted antiviral profile in the acute phase, whereas those with CCD exhibit a broad, non-resolving inflammatory signature. Across analyses, higher IL-23 and RA diagnosis tracked with persistence, while FGF-2, IL-4, and IgG seropositivity aligned with earlier resolution. These data identify candidate biomarkers and mechanistic pathways that warrant validation and may inform prevention and management strategies for chronic chikungunya disease.

## Supplementary Information

Below is the link to the electronic supplementary material.

Supplementary Material 1

## Data Availability

All essential data is presented on paper or appended as supplementary data. Additional data could be obtained from the corresponding authors upon requisition.
