# Supplementary material for: Distinct inflammatory biomarkers associated with rheumatological outcomes in chronic chikungunya disease
Source: Sci Rep. 2026 Apr 17;16:18469. doi: 10.1038/s41598-026-35570-x (PMC13266049; doi:10.1038/s41598-026-35570-x)
Supplement: Supplementary file 1 — Supplementary Material 1 [file 41598_2026_35570_MOESM1_ESM.docx]

**Persistent chronic chikungunya leading to rheumatological diseases in patients is associated with a robust inflammatory profile**

Lucas Sousa Magalhães, Juliana Cardoso Alves, Regina Adalva de Lucena Couto Ócea, Alejandra Debbo, Priscila Lima dos Santos, Suresh Mahalingam, Mauro Martins Teixeira, Amélia Maria Ribeiro de Jesus, Angela Maria da Silva, Roque Pacheco de Almeida, Camilla Natália Oliveira Santos

| **Supplementary Table 1** Summary of the multiple linear regression for clinical characteristics obtained during among case group (Chronic-CHIKV) and | | | | | |
| --- | --- | --- | --- | --- | --- |
| Predictor | β estimate | Standard Error | t-Value | p-value | 95% CI (lower; upper) |
| (Intercept) | -0.555 | 0.447 | -1.244 | 0.222 | -1.457; 0.347 |
| Age (years) | 0.013 | 0.0045 | 2.927 | 0.0059 | 0.004; 0.022 |
| VAS (0–10) | 0.131 | 0.0519 | 2.531 | 0.0159 | 0.027; 0.235 |
| n = 40  R² = 0.450 (Adjusted R² = 0.405)  Residual standard error = 0.391 (df = 36)  F(2, 37) = 9.85, p < 0.0001 | | | | | |


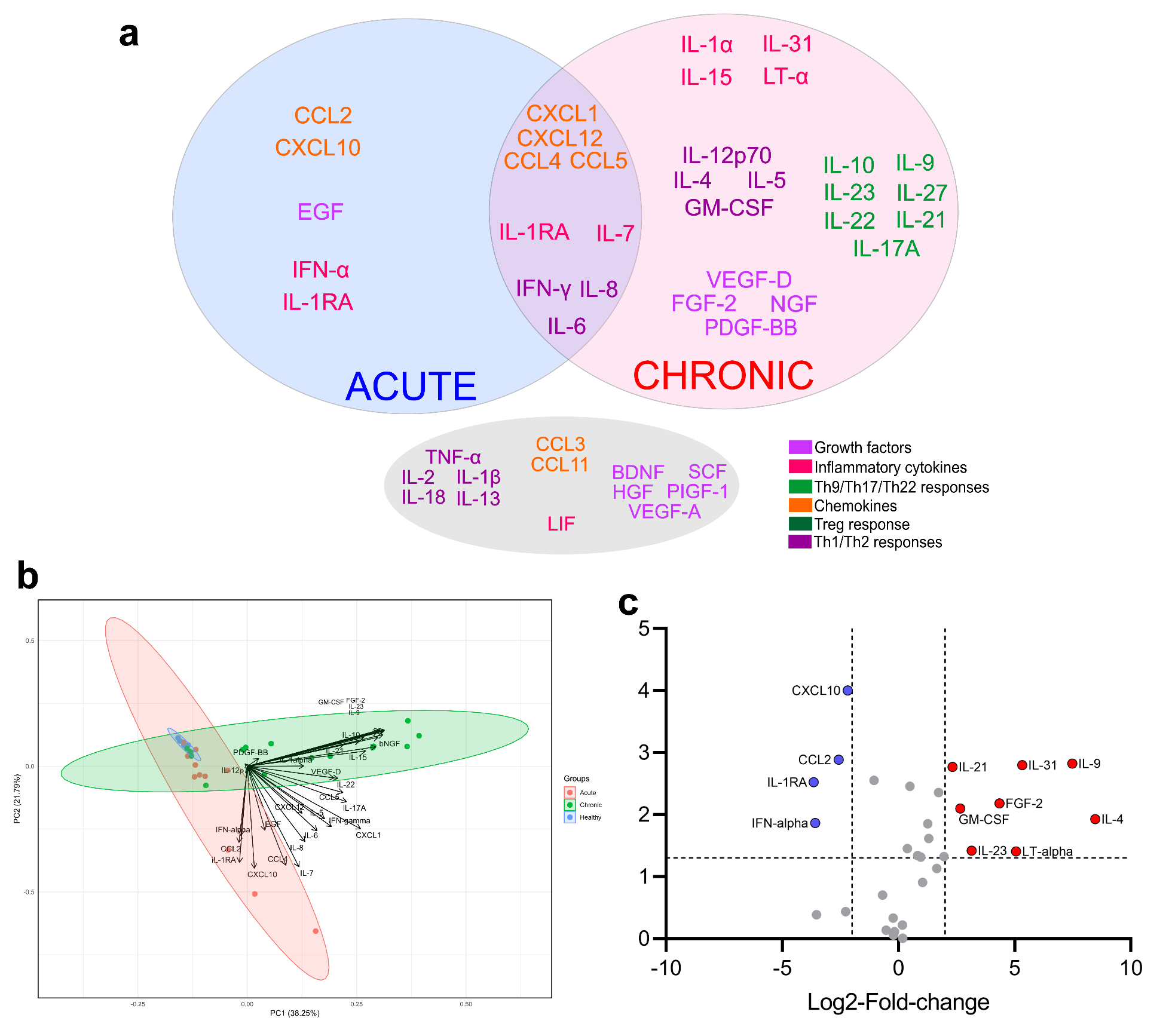


**Supplementary Figure 1** Diagram summarizing the results of measurement of the 45 analytes. Each circle includes immune mediators with higher levels in each group compared to the others: blue for people in Acute-chikungunya group, Chronic-chikungunya people in red, and gray for mediators with no differences among groups. All differences were assessed using the Kruskal-Wallis test followed by Dunn's test with adjusted p-values.

**Supplementary Figure 2** Cytokines, chemokines, and growth factors quantified in the sera of persons with Acute (blue) and Chronic (red) chikungunya disease and Healthy donors (gray). Results showing mediators without differences among groups. Each dot represents a person, boxplots show median plus 25% and 75% interquartile range and error bars show minimum and maximum values. Comparisons made by Kruskal-Wallis followed by Dunn test (adjusted p-values).

**
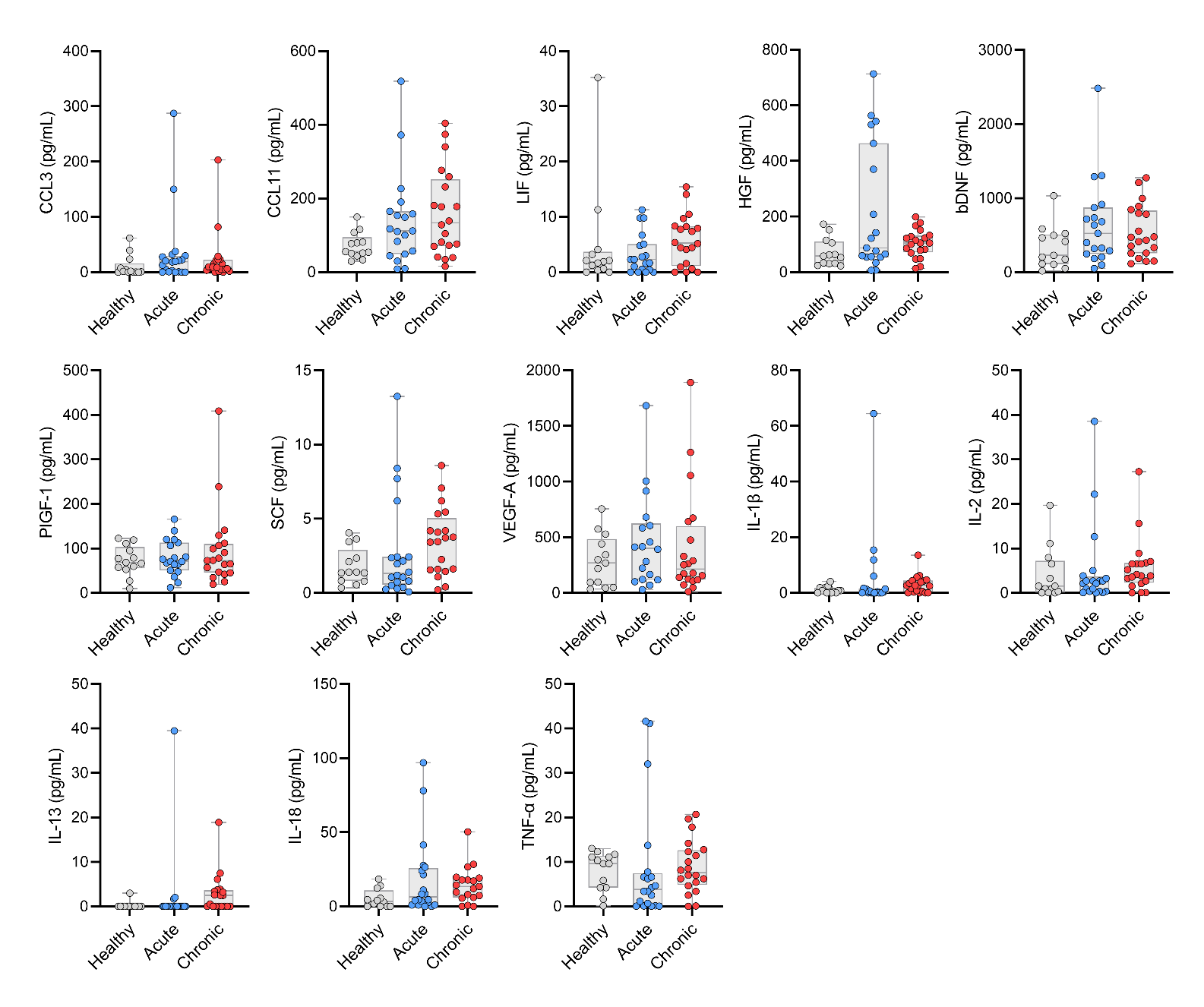
**
